# Experimental Study on Mechanical Properties of Desert Sand Concrete Under Freeze–Thaw Cycles

**DOI:** 10.3390/ma18071546

**Published:** 2025-03-29

**Authors:** Wenjie Xi, Zhiqiang Li, Yang Zhou, Gang Li, Feng Ji

**Affiliations:** 1 College of Water Conservancy & Architectural Engineering, Shihezi University, Shihezi 832003, China; 20222010005@stu.shzu.edu.cn (W.X.); zhougroup@shzu.edu.cn (Y.Z.); gangli@shzu.edu.cn (G.L.); 2 Academy of Water Sciences Co., Ltd., XPCC, Urumqi 830013, China; 3 College of Mathematics and Physics, Xinjiang Institute of Engineering, Urumqi 830023, China

**Keywords:** desert sand concrete, freeze–thaw cycle test, mechanical property, microstructure, freeze–thaw damage model

## Abstract

This study aims to explore the mechanical behavior of Desert Sand Concrete (DSC) under freeze–thaw (F-T) cycles. By adjusting the number of F-T cycles, the research analyzed the impact of various desert sand replacement ratios on the frost resistance of concrete. The study focused on the dynamic changes in mass loss of concrete specimens, relative dynamic elastic modulus, cubic compressive strength, splitting tensile strength, and axial compressive strength. Scanning electron microscopy was employed to analyze the micro-morphology of specimens after F-T cycles. This analysis aimed to predict the service life of DSC and provide practical recommendations for the maximum compressive strength loss rate within the designed service life. The results indicated that although the frost resistance of DSC was similar to that of ordinary concrete before 50 F-T cycles, it subsequently exhibited a nonlinear degradation trend correlated with increasing desert and replacement ratios, with both frost resistance and compactness reaching optimal levels at a 40% replacement rate. Additionally, the F-T damage model proposed in this study demonstrated high applicability and fitting accuracy. This model provided effective theoretical support for understanding and predicting the mechanical behavior of DSC.

## 1. Introduction

Evaluating the durability of concrete often involves examining its resistance to frost, which serves as a key performance metric [1]. In China’s northwest, north, and northeast regions, winter is characterized by low temperatures and considerable daily temperature fluctuations. These conditions subject reinforced concrete structures to significant F-T damage. As a result, the structural integrity and longevity of concrete are severely compromised. Rapid urban development has led to a dramatic increase in the consumption of river sand for engineering projects. However, such overexploitation poses a significant ecological threat. It not only undermines environmental health but also intensifies the supply–demand conflict for this vital resource. Leveraging the plentiful desert sand available domestically as a substitute for river sand in concrete manufacturing could significantly mitigate the imbalance between supply and demand for river sand [2,3], reduce land desertification [4,5], and lower the cost of construction projects.

Extensive research into the mechanical characteristics of concrete made with desert sand has been undertaken by scholars worldwide. Li et al. [6,7,8] explored the seismic performance of beam–column components made of desert sand concrete. Zhang et al. [9] established the optimal mix proportion for desert sand ceramsite lightweight aggregate concrete through orthogonal experiments and subsequently investigated its physical and mechanical properties using the derived optimal formulation. Li et al. [10,11] explored the performance and structural attributes of concrete incorporating aeolian sand, revealing the underlying processes through which this sand influences the structural characteristics of concrete at different levels. Zhang et al. [12] explored the mix ratio and performance evaluation of full aeolian sand high-performance concrete. Zhou et al. [13] assessed how the inclusion of hybrid fibers influences the compressive strength of aeolian sand-based concrete. Studies concerning the durability of concrete subjected to F-T cycles have primarily focused on the material’s frost resistance properties [14,15]. Key properties examined include the dynamic elastic modulus in relative terms [16] and the compressive strength of cubic specimens. Additionally, efforts have been made to establish models for the evolution of concrete damage in response to F-T cycles. Wang et al. [17] investigated how the water–cement ratio affects the printability and mechanical characteristics of 3D-printed concrete made with desert sand. Their analysis delved into the correlation between F-T cycles and several key parameters, such as mass reduction, relative dynamic modulus of elasticity, and the level of interfacial porosity. Additionally, they constructed a degradation model for F-T damage under varying water–cement ratios. Zhang et al. [18] explored the durability of aeolian sand concrete from macroscopic and microscopic perspectives, combining multi-scale research methods. The study revealed that optimal frost resistance was achieved when aeolian sand completely replaced river sand. Dong et al. [19] utilized ultrasonic pulse velocity and resonant frequency measurements to assess F-T damage in concrete incorporating aeolian sand pumice as a lightweight aggregate. They found that the optimal replacement rate of aeolian sand for river sand ranged from 20% to 30% and developed a damage constitutive model. Bai et al. [20] revealed that aeolian sand could inhibit the deterioration process of concrete, enhancing its frost resistance and durability. They also established a F-T damage degradation model and verified its reliability.

Relevant research findings indicate that there have been some outcomes regarding the F-T damage mechanism in ordinary concrete. However, further in-depth exploration and refinement is still required for studying the post-F-T failure mechanism and mechanical models of DSC. In light of this, this study delves into the impact of diverse ratios of desert sand substitution and the effects of F-T cycles on the mechanical characteristics of concrete. Electron microscopy scanning technology is employed to study the microstructural characteristics of DSC. A F-T damage degradation model for DSC is then constructed, using the relative dynamic elastic modulus and relative compressive strength as indicators. The aim of this study is to establish a theoretical foundation and provide technical support for predicting the lifespan of DSC in the cold regions of northern China. This work seeks to promote the development and application of this material in engineering projects in severe cold areas.

## 2. Materials and Methods

### 2.1. Materials

**Figure 1:**
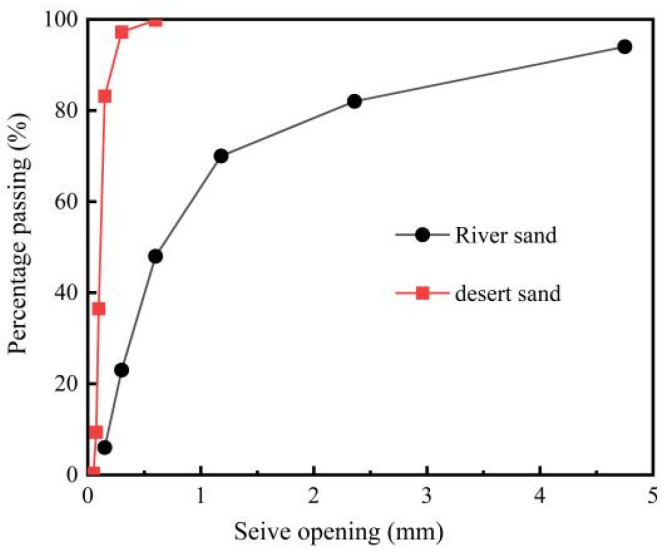
Particle size distributions of the sand utilized.

The experimental program required six principal material components for DSC formulation: fine aggregates (river sand and desert sand), coarse aggregate, cement, water-reducing admixture, and tap water. Table 1 summarizes the physicochemical properties and specifications of these constituents, while Figure 1 illustrates the particle size distribution analysis.

River sand, procured from the Manas River basin (Shihezi City, Xinjiang Province, China), was characterized as medium-grained washed sand with a fineness modulus of 2.58. In contrast, the desert sand exhibited a significantly lower fineness modulus of 0.198 (Table 1). Both fine aggregates complied with particle gradation requirements specified in JGJ 52 and ASTM C33 [21,22], as evidenced by their sieve analysis curves in Figure 1.

Crushed stone (5–26.5 mm nominal size) sourced from Shihezi City served as coarse aggregate. The binder system employed P·O 42.5 (R) ordinary Portland cement (Xinjiang Tianye Group) with documented setting characteristics: initial setting time = 2.7 h, final setting time = 4 h, and standard water demand = 28.8%. The cement demonstrated 28-day mechanical strengths of 7.8 MPa (flexural) and 48.8 MPa (compressive). A polycarboxylate-based high-performance water reducer (HSC type) achieving 20% water reduction was incorporated, with tap water completing the mixture formulation.

### 2.2. Mix Proportions

When the desert sand constitutes less than 60% of the mixture, the concrete exhibits good workability [6]. Therefore, this experiment utilizes desert sand replacement ratios, which are the percentages of the mass of desert sand to the mass of fine aggregate of 0%, 20%, 40%, and 60% for eight types of DSC. The strength design grade of the concrete is C40. Table 2 details the mix proportions and materials used for the concrete.

### 2.3. Method

#### 2.3.1. Freeze–Thaw Cycling

Four sets of prismatic specimens with dimensions of 100 mm × 100 mm× 400 mm were designed, with three specimens per group, to analyze mass and dynamic modulus of elasticity reduction. Additionally, four sets of cubic specimens with volumes of 100 mm × 100 mm × 100 mm were prepared, with six specimens per group, for compressive strength tests and splitting tensile strength tests under F-T cycling conditions. Four sets of prismatic specimens with dimensions of 100 mm × 100 mm × 300 mm were also designed, with three specimens per group, for axial compressive strength tests under F-T cycling conditions. The design parameters of the specimens are shown in Table 3.

After 24 h of moist curing, the specimens were demolded and subsequently maintained in a controlled temperature and humidity chamber for an additional 24 days, followed by a 4-day immersion in water to ensure adequate water absorption. This experiment utilized the TDRF-1 type rapid F-T testing machine for these specimens, following the guidelines set forth in the Chinese standard GB/T 50082 [23]. Each F-T cycle should last for 2 to 4 h, maintaining the central temperature of the specimen at −18 °C ± 2 °C and a maximum temperature of 5 °C ± 2 °C. The experiment is terminated if either of the following conditions occur:

- upon completion of the specified 200 F-T cycles;
- when the mass loss rate of the specimen exceeds or equals 5%;
- when the relative dynamic elastic modulus of the specimen drops below 60%.

The weight and dynamic modulus of elasticity of prismatic samples, each with dimensions of 100 mm × 100 mm × 400 mm, are assessed after each set of 25 F-T cycles. Cubic specimens are assessed for compressive strength and splitting tensile strength after every 50 cycles. The prismatic specimens with dimensions of 100 × 100 × 300 mm are tested for axial compressive strength after every 50 cycles.

#### 2.3.2. Macro Characterization

(1) Mass loss rate

The mass loss rate is one of the important indicators for assessing the F-T resistance of concrete. The mass loss rate, as indicated by Equation (1), is used to measure the quality changes in DSC under F-T cycling.

(1) $$\Delta W_{n}=\frac{W_{0}-W_{n}}{W_{0}}\times 100\%$$

where $\Delta W_{n}$ denotes the mass loss rate of the concrete specimen following *n* cycles of F-T, $W_{0}$ is the mass of the concrete specimen prior to the F-T test, $W_{n}$ represents the mass of the concrete specimen after *n* cycles of F-T.

(2) Relative dynamic elastic modulus

The relative dynamic elastic modulus provides a clear indication of the internal porosity of concrete and is a key parameter for evaluating F-T damage. The extent of damage in DSC is evaluated using the relative dynamic elastic modulus, as depicted in Equation (2).

(2) $$\Delta E_{n}=\frac{E_{n}}{E_{0}}\times 100\%=\frac{f_{n}^{2}}{f_{0}^{2}}\times 100\%$$

In the formula, $\Delta E_{n}$ represents the relative dynamic elastic modulus of the concrete specimen after *n* cycles of F-T, $E_{n}$ and $f_{n}$ are the dynamic elastic modulus and transverse natural frequency (measured through resonance frequency testing per Chinese standard GB/T 50082) of the DSC specimen following *n* F-T cycles, respectively, whereas $E_{0}$ and $f_{0}$ denote the initial dynamic elastic modulus and transverse natural frequency of the DSC specimen prior to F-T, respectively.

(3) Cube compressive strength loss rate

The loss rate of the cubic compressive strength is defined to assess the load-bearing capacity of DSC, as shown in Equation (3).

(3) $$\Delta f_{c1}=\frac{f_{c1,0}-f_{c1,n}}{f_{c1,0}}\times 100\%$$

In the equation, $\Delta f_{c1}$ represents the rate of compressive strength loss in the concrete specimen following *n* F-T cycles, $f_{c1,0}$ and $f_{c1,n}$ denote the cubic compressive strength of the concrete in its initial condition and after *n* cycles of F-T, respectively.

(4) Splitting tensile strength loss rate

The loss rate of the splitting tensile strength is defined to assess the crack resistance of DSC, as indicated in Equation (4).

(4) $$\Delta f_{t}=\frac{f_{t0}-f_{t1}}{f_{t0}}\times 100\%$$

In the formula, $\Delta f_{t}$ represents the loss rate of the splitting tensile strength of the concrete specimen following *n* F-T cycles, $f_{t0}$ and $f_{t1}$ denote the splitting tensile strength of the concrete in its initial state and after *n* F-T cycles, respectively.

(5) Axial compressive strength loss rate

In practical engineering, the length of compression members is often much greater than their cross-sectional dimensions. Hence, the axial compressive strength is a better indicator of the actual load-bearing ability of concrete than the cubic compressive strength. The definition of the axial compressive strength loss rate is presented in the following equation.

(5) $$\Delta f_{c2}=\frac{f_{c2,0}-f_{c2,n}}{f_{c2,0}}\times 100\%$$

In the equation, $\Delta f_{c2}$ denotes the loss rate of the axial compressive strength of the concrete specimen after *n* F-T cycles, $f_{c2,0}$ and $f_{c2,n}$ represent the axial compressive strength of the concrete in its original condition and following *n* F-T cycles, respectively.

#### 2.3.3. Microstructure

The apparent damage of concrete is often accompanied by changes in its microstructure. The evolution of the hydration products and the interfacial transition zone (ITZ) of DSC were characterized using a Zeiss Sigma 300 field-emission scanning (Carl Zeiss AG, Oberkochen City, Germany) electron microscope (SEM), and the microstructure of DSC under F-T conditions was evaluated.

## 3. Analysis of the Test Results

The macroscopic characterization of concrete after 200 F-T cycles is presented in Table 4.

### 3.1. Scaling Damage

**Figure 2:**
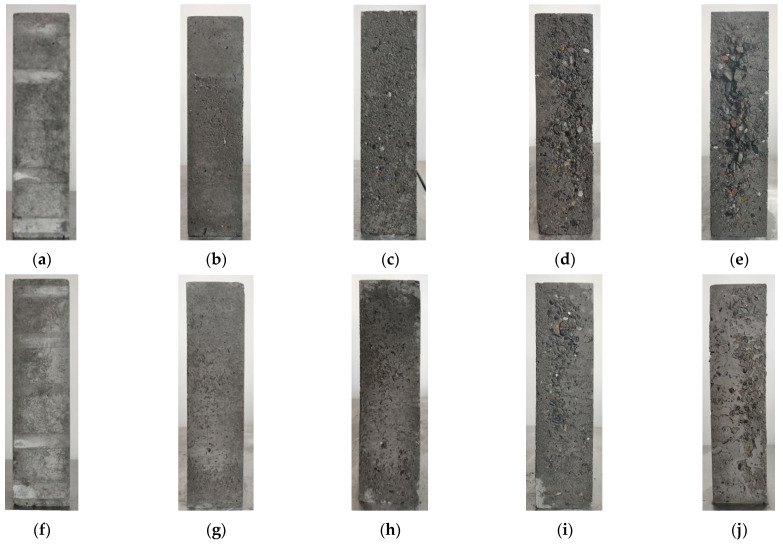
Apparent morphology of specimens under different freeze–thaw cycles: (**a**) NC-2, n = 0. (**b**) NC-2, n = 50. (**c**) NC-2, n = 100. (**d**) NC-2, n = 150. (**e**) NC-2, n = 200. (**f**) DSC-40-2, n = 0. (**g**) DSC-40-2, n = 50. (**h**) DSC-40-2, n = 100. (**i**) DSC-40-2, n = 150. (**j**) DSC-40-2, n = 200.

The changes in concrete specimens during F-T cycling are directly reflected in their surface morphology. This morphology, to some extent, reveals the F-T damage sustained by the concrete material. Under F-T action, significant alterations occur on the surface of both ordinary and desert sand concretes. The surface morphology of the NC-2 and DSC-40-2 groups of specimens after F-T cycling is shown in Figure 2.

As can be seen from Figure 2: in the initial state of the experiment, the cement paste tightly envelops the aggregates with a smooth surface; after 50 cycles of F-T, the concrete surface becomes uneven, with minor mortar detachment and the formation of fine pores. Following 100 F-T cycles, the surface of the specimens gradually becomes rougher. There is an increased number of fine, dense pores. Additionally, a small amount of coarse aggregates in the NC-2 group specimens begins to be exposed. After 150 F-T cycles, the exposure of coarse aggregates in the DSC-40-2 group specimens significantly increases. In contrast, the NC-2 group specimens show a greater amount of mortar spalling. Additionally, the exposure of coarse aggregates in the NC-2 group is more pronounced. By the 200th F-T cycle, the exposure of coarse aggregates in the DSC-40-2 group specimens has increased, but it remains uniformly distributed. In contrast, the NC-2 group specimens exhibit more severe spalling of coarse aggregates. Some aggregates are noticeably exposed. Compared to the DSC-40-2 group, the concrete in the NC-2 group exhibits denser cracks and more extensive surface damage. Additionally, the mortar in the damaged areas is in a powdery state and can be easily dislodged with a slight touch. Overall, under identical F-T cycling conditions, the deterioration of apparent damage in ordinary concrete is significantly greater than that in DSC.

### 3.2. Mass Loss Rate

**Figure 3:**
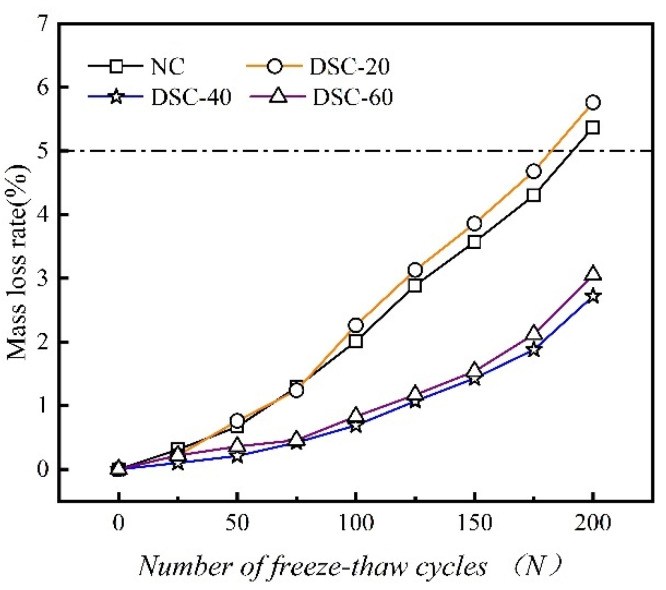
Variation curve of mass loss rate with the number of freeze–thaw cycles.

The variation curve between the mass loss rate and the number of F-T cycles is plotted, as shown in Figure 3.

The figure indicates that with the increase in F-T duration, the mass loss rate of both ordinary and DSC specimens tends to rise. Prior to 50 F-T cycles, the mass loss rate curves of the ordinary and desert sand groups exhibit similar developmental patterns. All concrete groups reach a state of water saturation before the rapid onset of F-T cycles. Consequently, the early mass loss is not significant. Following 200 F-T cycles, the mass loss rates of specimens NC-2, DSC-20-2, DSC-40-2, and DSC-60-2 are 5.37%, 5.76%, 2.72%, and 3.05%, respectively. The specimens of groups NC-2 and DSC-20-2 have reached the mass loss criteria for damage, while groups DSC-40-2 and DSC-60-2 exhibit superior freeze–thaw resistance. Furthermore, the mass loss rate of the DSC-20-2 group exceeds that of ordinary concrete after 75 F-T cycles. This effect is attributed to the small particle size of desert sand. When combined with river sand and coarse aggregates, it creates an excellent gradation system. This gradation system enhances the compactness of the concrete structure. However, concrete with a 20% desert sand replacement ratio produces a large number of fine, enclosed pores [11]. These pores lack the capacity to endure internal F-T stresses in the concrete, resulting in heightened F-T damage.

### 3.3. Relative Dynamic Elastic Modulus

**Figure 4:**
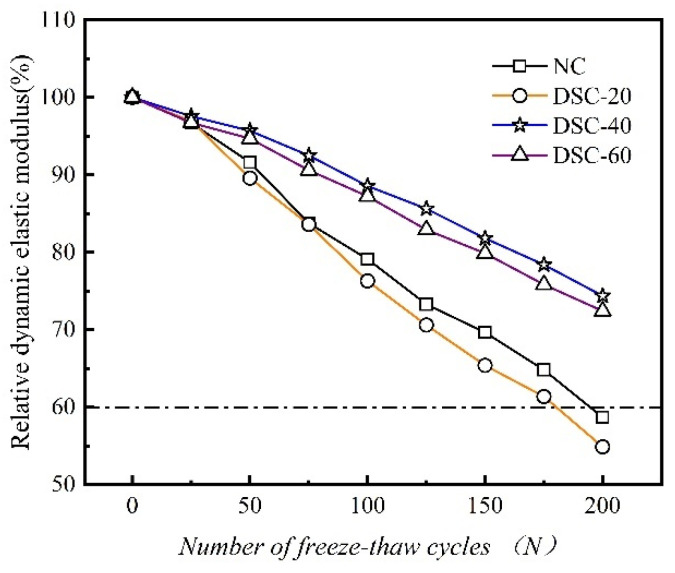
The variation curves of relative dynamic elastic modulus as a function of the number of freeze–thaw cycles.

Figure 4 depicts the correlation between the dynamic elastic modulus in relative terms and the count of F-T cycles.

Figure 4 indicates that as the number of F-T cycles increases, the relative dynamic elastic modulus of both ordinary and desert sand concrete specimens shows a declining trend. This phenomenon can be attributed to the porous structure resulting from hydrostatic pressure [24] and osmotic pressure [25]. At the onset of F-T cycling, the phase change in water within the concrete pores induces volumetric expansion. This expansion leads to surface spalling. As the quantity of F-T cycles accumulates, internal damage within the concrete progressively intensifies, exacerbating structural degradation. Initially, during the early stages of F-T, there is no notable difference in the variation in dynamic elastic modulus among the groups. However, following 50 cycles, a more pronounced decrease in the relative dynamic elastic modulus is observed in the NC-2 and DSC-20-2 groups. In contrast, the DSC-40-2 and DSC-60-2 groups begin to show divergence. Upon completing 200 F-T cycles, the concrete’s relative dynamic elastic modulus is 0.59, 0.55, 0.74, and 0.72, respectively. The damage degree to the relative dynamic elastic modulus exhibits a biphasic response to desert sand replacement rates: initial mitigation (≤20% replacement) followed by progressive exacerbation beyond this threshold. Both the NC-2 and DSC-20-2 groups experienced significant damage. This damage includes the continuous increase and propagation of internal micro-cracks. The relative dynamic elastic modulus of these groups meets the failure criteria. The expansion and osmotic pressures within the capillary pores of the DSC increase, causing damage to its internal structure. This results in a marked reduction in the relative dynamic elastic modulus, aligning with findings reported in the literature [26].

### 3.4. Cube Compressive Strength Loss Rate

**Figure 5:**
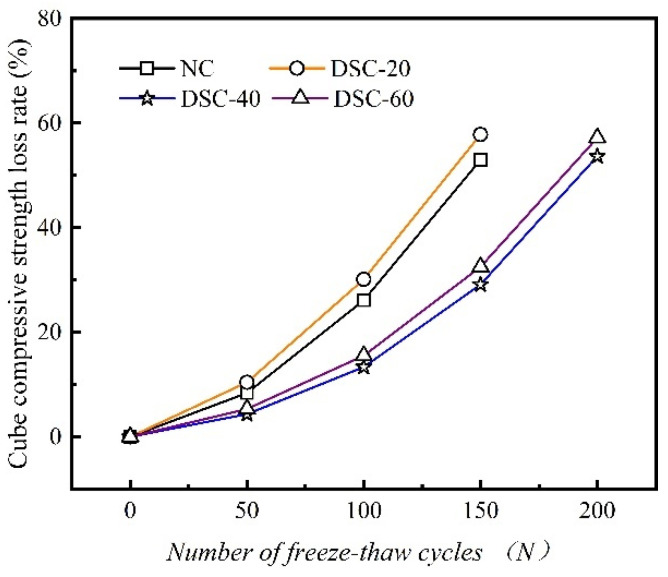
The relationship between cubic compressive strength loss rate and the number of freeze–thaw cycles.

The variation curves depicting the loss rate of cubic compressive strength in concrete, corresponding to various desert sand replacement ratios, are illustrated as a function of the number of F-T cycles. These curves are shown in Figure 5.

Figure 5 illustrates that the cubic compressive strength of all concrete specimens progressively diminishes with the increasing number of F-T cycles. Furthermore, the rate of decrease becomes progressively more significant. As the number of F-T cycles remains constant, the compressive strength trend initially shows a decline, followed by an increase, and then a subsequent decrease as the desert sand replacement ratio rises. When the number of F-T cycles remains below 50, the rate of decrease in compressive strength is relatively slow. This indicates that the concrete experiences less severe freeze–thaw damage during the early stages. After 50 F-T cycles, the slope of the compressive strength decline increases. This is primarily because, in the later stages of F-T cycling, internal damage within the concrete accumulates continuously. As a result, there is a rapid decline in concrete strength. Furthermore, after 150 F-T cycles, the compressive strengths of the four groups of DSC with different mix proportions are as follows: 20.77 MPa, 17.19 MPa, 32.38 MPa, and 29.5 MPa. These values represent decreases of 53.0%, 57.8%, 29.1%, and 32.5% from their initial strengths, respectively. The NC and DSC-20 specimens, after undergoing 200 freeze–thaw cycles, were too damaged to measure their compressive strength values. This indicates that when the desert sand replacement ratio is 40% and 60%, the cubic compressive strength of the specimens subjected to F-T cycles performs better than that of ordinary concrete. It is noteworthy that when the desert sand content is 20%, the compressive strength of the specimens is lower than that of ordinary concrete. This reduction is primarily due to the lower replacement ratio, which diminishes the advantage of angular particles. Consequently, the aggregate’s resistance to cracking is reduced.

### 3.5. Splitting Tensile Strength Loss Rate

**Figure 6:**
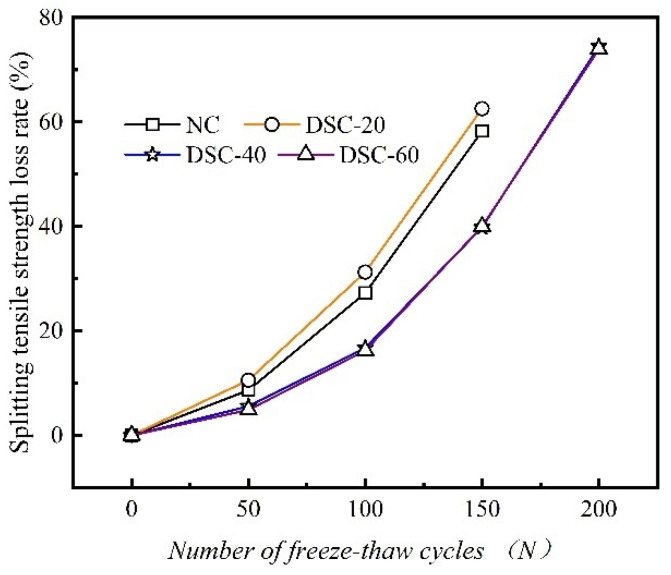
Relationship between splitting tensile strength loss rate and number of freeze–thaw cycles.

Figure 6 illustrates the variation curves of the splitting tensile strength of concrete with various desert sand replacement ratios in relation to the number of F-T cycles.

Figure 6 demonstrates that the variation pattern of the splitting tensile strength of concrete parallels that of its compressive strength. Nevertheless, the splitting tensile strength shows a significantly more pronounced decline as the number of F-T cycles increases. Under the same number of F-T cycles, the specimens from the DSC-20-2 group exhibit the greatest reduction in splitting tensile strength. This is followed by ordinary concrete, while the DSC-40-2 group specimens show the smallest decrease in strength. After 150 F-T cycles, the splitting tensile strengths for both conventional concrete and concrete with desert sand, at varying substitution levels, were recorded at 1.01 MPa, 0.85 MPa, 1.51 MPa, and 1.59 MPa, respectively. These values represent decreases of 58.26%, 62.56%, 39.84%, and 40.0%, respectively, from their initial conditions. After 200 F-T cycles, the specimens from the NC-2 and DSC-20-2 groups exhibit significant damage and compromised integrity. Therefore, they are not suitable for splitting tensile strength tests. By comparing the slopes of the four curves shown in the figure, it is observed that the curve corresponding to the DSC-20-2 group specimens has the largest absolute slope value. This indicates that the F-T action exerts the most pronounced effect on this group of specimens. This is due to the fact that F-T action damages the aggregate–cement paste interface. Additionally, a low proportion of desert sand introduces more defects into the concrete. In particular, when the concrete is subjected to load, cracks tend to initiate along the aggregate-paste interface [27]. The ratio of the splitting tensile strength to the compressive strength of DSC fluctuates within a small range around 0.054.

### 3.6. Axial Compressive Strength Loss Rate

**Figure 7:**
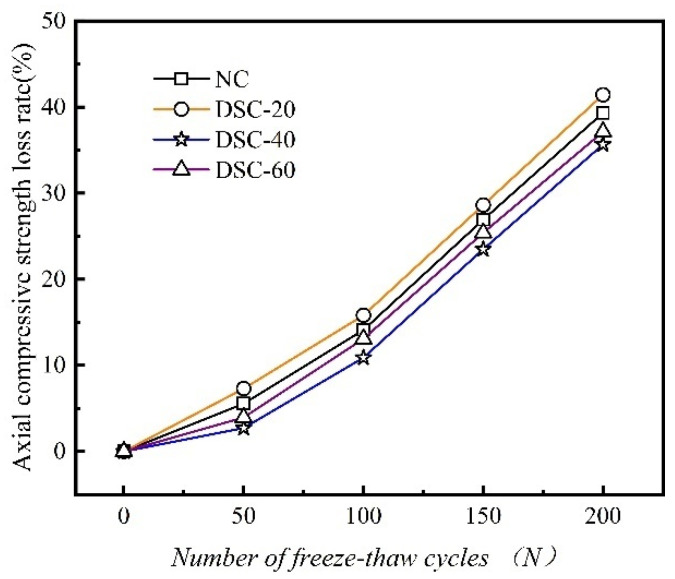
Variation curve of axial compressive strength with the number of freeze–thaw cycles.

The axial compressive strength more accurately reflects the actual performance of structural members than the cubic compressive strength. The variation curves of the axial compressive strength loss rate for ordinary and desert sand concretes under F-T action are presented in Figure 7.

As shown in Figure 7, both ordinary and desert sand concrete exhibit a gradual decline in axial compressive strength as the F-T duration increases. This decline is accompanied by an accelerating trend in deterioration. Under the same F-T duration, the axial compressive strength of DSC initially decreases as the desert sand replacement ratio increases. This is followed by an increase in strength, which is then succeeded by a subsequent decrease with further increases in the replacement ratio. Following 150 F-T cycles, the axial compressive strengths of DSC with replacement ratios from NC to DSC-60 are 21.74 MPa, 19.78 MPa, 24.19 MPa, and 22.30 MPa, respectively. These values represent decreases of 26.92%, 28.62%, 23.47%, and 25.39% from their pre-freeze–thaw values. The axial compressive strength of the DSC-20-3 group specimens is inferior to that of ordinary concrete, while the DSC-40-3 and DSC-60-3 groups exhibit higher axial compressive strengths than ordinary concrete. Notably, the DSC with a 40% replacement ratio achieves the highest axial compressive strength. This is primarily because the addition of desert sand fills the micro-gaps within the concrete. This results in a denser and more uniform structure that better resists internal freeze–thaw stresses. Consequently, crack propagation during the F-T process is slowed down. However, due to the smooth surface of desert sand particles, a low desert sand replacement ratio can reduce the bond strength between the mortar and aggregates. This results in a reduction in the axial compressive strength of the specimens following F-T cycles.

### 3.7. Microscopic Characterization

**Figure 8:**
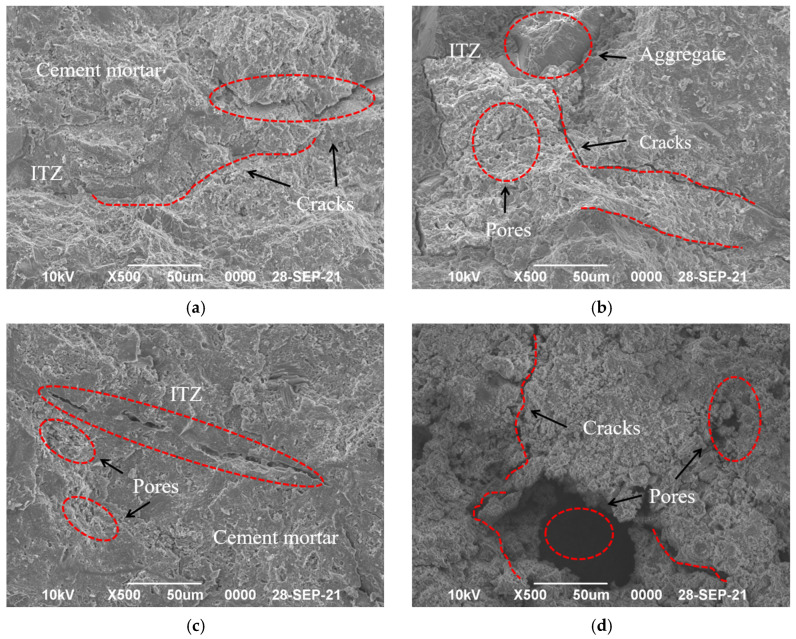
Microstructure before and after freeze–thaw: (**a**) NC, n = 0. (**b**) NC, n = 200. (**c**) DSC-40, n = 0. (**d**) DSC-40, n = 200.

The apparent damage of concrete is often accompanied by changes in its microstructure. To elucidate the freeze–thaw damage mechanism of DSC, two representative groups of concrete specimens were selected for scanning electron microscopy (SEM) analysis [28,29]. SEM tests were conducted on specimens from both the NC group and the DSC-40 group. These tests were performed to comparatively analyze changes in the internal structure of the specimens. The results are illustrated in Figure 8.

Figure 8 illustrates that prior to the F-T cycling, the interfacial transition zone (ITZ) structure of all specimens was essentially intact. Under low magnification, no apparent cohesive cracks were observed at the aggregate–mortar interface, and the overall structure was in good condition. The NC specimens exhibited a few micro-cracks and closed small pores, with no significant initial cracks observed at the interface of the coarse aggregate and the cement mortar. After 200 F-T cycles, the ITZ structure of the specimens showed significant deterioration. The internal structure became looser, with noticeable separation between the aggregate and mortar phases. There was an increase in the number of internal pores and more pronounced cohesive cracks. The mortar matrix in the NC specimens became noticeably friable and brittle. Clear cohesive cracks extended into the mortar matrix, leading to mortar detachment and spalling. This resulted in the formation of larger pits and a decline in overall integrity, indicating insufficient frost resistance. As shown in Figure 8d, the aggregate is more tightly bonded with the matrix. However, fine cracks appeared in the ITZ, extending into the matrix, which began to detach. Despite this, the degree of damage was relatively minor, demonstrating better macroscopic frost resistance. This improvement is primarily due to the incorporation of an appropriate amount of desert sand. The inclusion of this material changed the makeup of the cementitious mortar, thereby enhancing the adhesive force between the mortar and the aggregates.

## 4. Freeze–Thaw Damage Model of Concrete

Macroscopic concrete damage, including variations in mass, relative dynamic elastic modulus, compressive strength, and tensile strength, signifies the internal damage accumulation within the material. The dynamic elastic modulus loss model effectively captures the concrete’s damage state and offers ease of measurement. Given that some specimens were already damaged after 150 freeze–thaw cycles and considering that concrete structures primarily endure pressure in engineering applications, this study establishes a F-T cycle action model for desert sand concrete. This model is based on data from the first 150 F-T cycles, using the relative dynamic elastic modulus and relative compressive strength as indicators. This model allows for effective prediction of the service life of desert sand concrete in northern regions under F-T conditions. It provides important reference and guidance for its application in harsh environments.

### 4.1. Dynamic Elastic Modulus Damage Attenuation Model

Regarding the dynamic elastic modulus damage decay model, this study proposes a degree of damage for DSC, as shown in Equation (6):

(6) $$D_{E}=1-\frac{E_{N}}{E_{0}}$$

In the equation, $D_{E}$ represents the degree of dynamic elastic modulus damage of the concrete after F-T cycling; where $D_{E}$ ≤ 1; $E_{0}$ denotes the initial dynamic elastic modulus of the specimen at the start of the F-T test; $E_{N}$ is the dynamic elastic modulus of the concrete specimen after *n* cycles of F-T.

Based on the literature [17,30] and incorporating dynamic elastic modulus data from the F-T cycle tests conducted in this study, a F-T damage model for DSC has been formulated. This model is represented by Equation (7):

(7) $$D_{E}=AN^{2}+BN+C$$

where *N* signifies the number of freeze–thaw cycles; coefficients *A*, *B*, and *C* pertain to the damage model for the dynamic modulus of elasticity under F-T cycling conditions.

**Figure 9:**
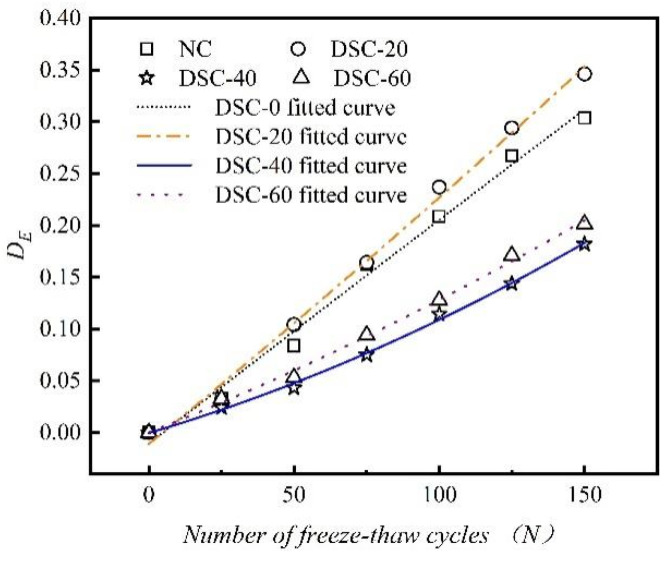
Variation curve of dynamic elastic modulus freeze–thaw damage with the number of freeze–thaw cycles.

The variation in dynamic elastic modulus damage and the corresponding fitting curves for concrete with different desert sand replacement ratios are illustrated in Figure 9.

As shown in Figure 9, the fitted curves illustrating F-T damage evolution exhibit a quadratic functional relationship. Additionally, the freeze–thaw damage and degradation rate of concrete containing an appropriate amount of desert sand are significantly lower than those observed in ordinary concrete. It is evident that a desert sand replacement ratio of 40% can significantly slow down the degradation rate of DSC. The data points match the fitting curves with a high degree of accuracy. Table 5 presents the fitting parameters for the elastic modulus damage degree of concrete at different desert sand replacement ratios. All correlation coefficients surpass 0.991, demonstrating a high degree of fitting accuracy.

### 4.2. Compressive Strength Damage Attenuation Model

Regarding the compressive strength damage decay model, the degree of damage $D_{f1}$ for DSC is defined, as shown in Equation (8):

(8) $$D_{f1}=1-\frac{f_{c1}}{f_{c0}}$$

In the equation, $D_{f1}$ represents the degree of compressive strength damage in concrete subjected to F-T cycles, where $D_{f1}$ ≤ 1.

**Figure 10:**
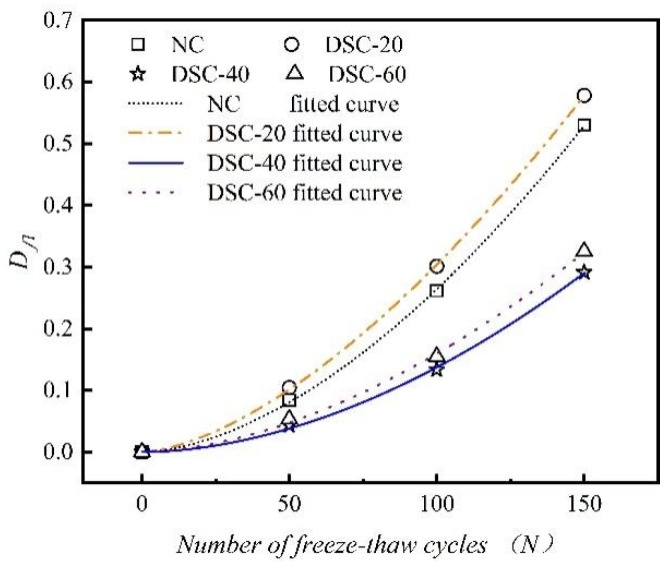
The curve of compressive strength frost damage varies with the number of freeze–thaw cycles.

Regarding the relative compressive strength freeze–thaw damage decay model, Wang et al. [31], in the context of recycled concrete under F-T action, established a formula for fitting its compressive strength in exponential form. Gan et al. [32] developed a decay model for sulfate erosion and freeze–thaw damage based on a cubic function form. By comparison, it was concluded that the accuracy of the aforementioned damage prediction models in fitting the data from this experiment is not high. Building on this and incorporating the compressive strength data obtained from the F-T action in this experiment—particularly for desert sand concrete—a predictive calculation model for the decay of compressive strength under F-T conditions is proposed. This model is represented as a power function, illustrated in Equation (9):

(9) $$D_{f1}=aN^{b}$$

where N signifies the number of freeze–thaw cycles; a and b are the power function coefficients for the decay of compressive strength during F-T cycling. Figure 10 illustrates the changes in compressive strength damage and the associated fitting curves for concrete with varying ratios of desert sand replacement.

Table 6 presents the fitting parameters related to the compressive strength damage of concrete incorporating various desert sand replacement ratios.

**Figure 11:**
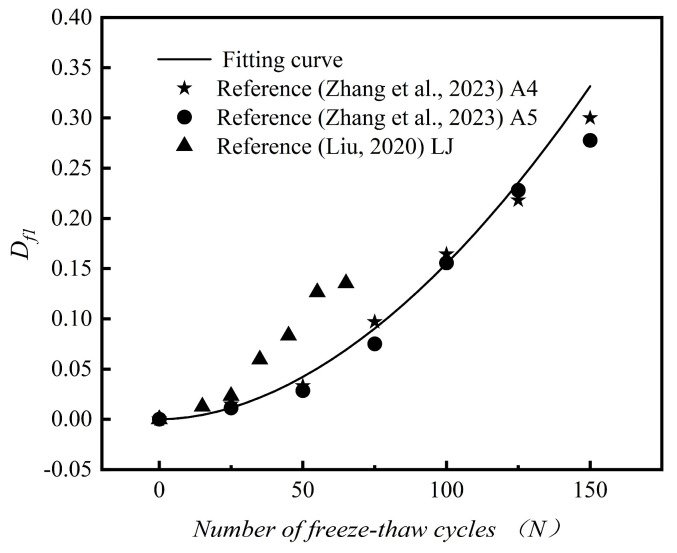
Comparison of the amount of compressive strength damage. Damage quantification of concrete compressive strength. Zhang et al. [18]; Liu [33]. All referenced studies are listed in the References section.

As evidenced by Table 6, the damage evolution models of concrete specimens incorporating varying desert sand replacement ratios exhibited correlation coefficients (R²) exceeding 0.998, demonstrating excellent fitting precision and thereby validating the appropriateness of employing a power function to characterize their compressive strength degradation mechanisms. To assess the rationality of the established model, a comparative analysis was conducted. This analysis compared the fitting curve of the DSC-40-3 group specimens, as obtained from the model, with the specimens from Groups A4 and A5 in the literature [18], as well as with the LJ group from the literature [33]. Figure 11 displays the findings of this comparison. It was observed that the data points from the relevant literature are scattered around the fitting curve. Compared with traditional dynamic elastic modulus damage decay models, this model more intuitively reflects the mechanical properties of DSC. As a result, it is more suitable for evaluating actual desert sand concrete.

### 4.3. Predicting the Lifespan of Desert Sand Concrete Under Freeze–Thaw Cycles

In accordance with the definition of concrete frost resistance from the literature [34], this study calculates the limiting number of F-T cycles associated with a dynamic elastic modulus damage degree of 0.4. The computation utilizes Equation (7), with the outcomes displayed in Table 7.

Li et al. [35] summarized the number of F-T cycles affecting concrete in various regions of the country, considering both indoor and outdoor conditions. This summary is presented in Equation (10):

(10) $$T_{a}=kN/M$$

In the equation, $T_{a}$ represents the actual service life of the concrete, k is the F-T test coefficient for concrete, typically taken as 12, meaning that 12 rapid indoor F-T cycles are equivalent to 1 outdoor natural F-T cycle, N is the number of F-T cycles required to reach a damage degree of 0.4, M refers to the average number of F-T cycles that the concrete may experience within a year.

Taking into account the distribution of deserts and cold regions, and referencing the study by Wu et al. [36], the annual average number of F-T cycles in 15 representative areas in northwest, north, and northeast China was identified. Using Table 7, the anticipated service life for these typical regions was computed with Equation (10). The findings are illustrated in Table 8.

Table 8 illustrates that incorporating a suitable amount of desert sand (40% to 60%) markedly enhances the F-T durability of concrete. Specifically, DSC-40 concrete in the Urumqi region can achieve a service life of nearly 30 years. This discovery offers a theoretical basis for the practical application and promotion of desert sand concrete.

To further investigate the impact of different desert sand replacement ratios on the durability and compressive strength loss of concrete under freeze–thaw conditions, this study combines data from Table 7 and Table 8. Calculations are performed based on the compressive strength damage prediction model outlined in Section 4.2. The outcomes are displayed in Table 9.

As can be seen from Table 9, when reaching its predicted service life, concrete with an appropriate desert sand replacement ratio (40% to 60%) exhibits a lower compressive strength loss rate compared to ordinary concrete. DSC-40 exhibits the longest service life due to its higher initial compressive strength and denser internal structure. It also shows a relatively low cubic compressive strength loss rate of 17.48%.

## 5. Conclusions

This study integrates macroscopic mechanical performance tests to examine the surface morphology, mechanical properties, and microstructure of concrete with varying desert sand replacement ratios under F-T cycles. This research aims to establish a model for F-T damage in DSC and to offer a foundation for predicting its service life. The conclusions of this study are as follows:

(1) After F-T cycling, the trend of mass loss rate is essentially similar to that of the dynamic elastic modulus. As the number of F-T cycles increases, the damage to the specimens progressively worsens. After 175 cycles, the specimens from the NC and DSC-20 groups exhibit severe damage.
(2) The F-T resistance of DSC exhibits a trend that begins with an increase, is succeeded by a decline, and then experiences another rise with the progressive augmentation of the desert sand substitution ratio. The concrete achieves optimal F-T resistance at a 40% replacement rate. After 150 F-T cycles, the cubic compressive strength loss, splitting tensile strength loss, and axial compressive strength loss for the DSC-40 group specimens are 29.1%, 39.8%, and 23.5%, respectively.
(3) Scanning electron microscopy (SEM) was performed on both ordinary concrete and desert sand concrete (DSC) specimens after F-T cycling. The observations indicated that desert sand effectively occupies the pores in cement mortar. At a replacement ratio of 40%, the microstructure of specimens subjected to F-T cycles shows considerable enhancement.
(4) This study established a power function model to describe F-T damage decay based on relative compressive strength. The model achieved a fitting accuracy exceeding 0.998, demonstrating superior fitting effectiveness compared to the dynamic elastic modulus model. This model can effectively reflect the changes in mechanical properties and the extent of damage in DSC under the action of F-T cycles.
(5) This study focuses on the typical cold regions of northwest, north, and northeast China. It analyzes the F-T durability of concrete with varying desert sand replacement ratios. Among them, DSC-40 exhibits the highest F-T durability, reaching up to 44 years of life. At the predicted service life, the calculated compressive strength loss rates, based on the compressive strength damage model, are in the order of DSC-60 < DSC-40 < NC < DSC-20.

## Figures and Tables

**Table 1:** Physical property index of fine aggregate.

Fine Aggregate Category	Fineness Modulus/ kg·m^−3^	Apparent Density/ kg·m^−3^	Bulk Density/ kg·m^−3^	Mud Content/%	Water Absorption/%
Dune sand	0.198	2630	1615	1.9	2.1
River sand	2.58	2038	1350	2.2	0.8

**Table 2:** Mix proportion and DSC material consumption.

Specimens	Water–Binder Ratio	Sand Rate	Material Consumption/kg·m^−3^					
			Water	Cement	Water Reducer	Coarse Aggregates	River Sand	Dune Sand
NC	0.4	0.3	160	400	1.6	1288	552.0	0
DSC-20	0.4	0.3	160	400	1.6	1288	441.6	110.4
DSC-40	0.4	0.3	160	400	1.6	1288	331.2	220.8
DSC-60	0.4	0.3	160	400	1.6	1288	220.8	331.2

Note: DSC refers to Desert Sand Concrete, and NC denotes Normal Concrete, serving as the control group for the experiment.

**Table 3:** Specimen design parameters.

Specimen Number	Desert Sand Substitution Rate	Specimen Size			Number of Freeze–Thaw Cycles n	Number of Test Blocks
		Length/ mm	Height/ mm	Width/ mm		
NC-1	0	100	100	100	0, 50, 100, 150, 200	30
NC-2		100	100	400		3
NC-3		100	100	300	0, 50, 100, 150	12
DSC-20-1	20	100	100	100	0, 50, 100, 150, 200	30
DSC-20-2		100	100	400		3
DSC-20-3		100	100	300	0, 50, 100, 150	12
DSC-40-1	40	100	100	100	0, 50, 100, 150, 200	30
DSC-40-2		100	100	400		3
DSC-40-3		100	100	300	0, 50, 100, 150	12
DSC-60-1	60	100	100	100	0, 50, 100, 150, 200	30
DSC-60-2		100	100	400		3
DSC-60-3		100	100	300	0, 50, 100, 150	12

**Table 4:** Relative indices of concrete during 0–200 freeze–thaw damage.

No.	Test Project	0	25	50	75	100	125	150	175	200
NC	Δ*W_n_* (%)	0	0.31	0.67	1.29	2.01	2.89	3.57	4.3	5.37
	Δ*E_n_* (%)	100	96.71	91.61	83.75	79.12	73.28	69.65	64.8	58.68
	Δ*f_c1_* (%)	0	-	8.40	-	26.14	-	52.99	-	80.87
	Δ*f_t_* (%)	0	-	8.68	-	27.27	-	58.26	-	86.78
	Δ*f_c2_* (%)	0	-	5.55	-	14.12	-	26.92	-	39.33
DSC-20	Δ*W_n_* (%)	0	0.22	0.76	1.24	2.26	3.13	3.86	4.68	5.76
	Δ*E_n_* (%)	100	96.93	89.58	83.58	76.3	70.59	65.38	61.36	54.86
	Δ*f_c1_* (%)	0	-	10.43	-	30.13	-	57.83	-	84.45
	Δ*f_t_* (%)	0	-	10.57	-	31.28	-	62.56	-	89.43
	Δ*f_c2_* (%)	0	-	7.29	-	15.81	-	28.62	-	41.43
DSC-40	Δ*W_n_* (%)	0	0.1	0.21	0.42	0.69	1.07	1.43	1.88	2.72
	Δ*E_n_* (%)	100	97.58	95.68	92.49	88.56	85.62	81.8	78.38	74.37
	Δ*f_c1_* (%)	0	-	4.33	-	13.35	-	29.13	-	53.67
	Δ*f_t_* (%)	0	-	5.58	-	16.73	-	39.84	-	74.50
	Δ*f_c2_* (%)	0	-	2.72	-	10.88	-	23.47	-	35.65
DSC-60	Δ*W_n_* (%)	0	0.22	0.36	0.46	0.83	1.17	1.54	2.12	3.05
	Δ*E_n_* (%)	100	96.73	94.68	90.59	87.23	82.9	79.86	75.83	72.41
	Δ*f_c1_* (%)	0	-	5.33	-	15.51	-	32.53	-	57.20
	Δ*f_t_* (%)	0	-	4.91	-	16.23	-	40	-	73.96
	Δ*f_c2_* (%)	0	-	3.95	-	13.08	-	25.39	-	37.14

**Table 5:** The correlation coefficient of the dynamic elastic modulus damage attenuation model.

Specimen Number	A	B	C	Correlation Coefficient
NC-2	−2.019 × 10^−7^	2.18 × 10^−3^	−1.052 × 10^−2^	0.9915
DSC-20-2	9.638 × 10^−7^	2.28 × 10^−3^	1.088 × 10^−2^	0.9947
DSC-40-2	2.606 × 10^−6^	8.326 × 10^−4^	−3.714 × 10^−4^	0.9978
DSC-60-2	1.674 × 10^−6^	1.11 × 10^−3^	3.571 × 10^−5^	0.9969

**Table 6:** The correlation coefficient of the compressive strength damage attenuation model.

Specimen Number	*a*	*b*	Correlation Coefficient
NC-3	9.937 × 10^−5^	1.712	0.9981
DSC-20-3	2.071 × 10^−4^	1.583	0.9998
DSC-40-3	2.810 × 10^−5^	1.845	0.9987
DSC-60-3	5.181 × 10^−5^	1.744	0.9986

**Table 7:** The ultimate number of freeze–thaw cycles for DSC.

Specimen Number	NC	DSC-20	DSC-40	DSC-60
Ultimate number Of F-T cycles	192	160	264	259

**Table 8:** Frost durability life of DSC.

Region	Area	Average Annual Number of Freeze–Thaw Cycles	NC	DSC- 20	DSC- 40	DSC- 60
Northwestern China	Urumqi	107	21.5	17.9	29.6	29.0
	Xining	117	19.7	16.4	27.1	26.6
	Lanzhou	93	24.8	20.6	34.1	33.4
	Hohhot	120	19.2	16.0	26.4	25.9
	Yinchuan	106	21.7	18.1	29.9	29.3
Northern China	Shijiazhuang	73	31.6	26.3	43.4	42.6
	Taiyuan	100	23.0	19.2	31.7	31.1
	Beijing	84	27.4	22.9	37.7	37.0
	Tianjin	77	29.9	24.9	41.1	40.4
Northeast China	Mudanjiang	128	18.0	15.0	24.8	24.3
	Changchun	118	19.5	16.3	26.8	26.3
	Harbin	125	18.4	15.4	25.3	24.9
	Yanji	127	18.1	15.1	24.9	24.5
	Shenyang	105	21.9	18.3	30.2	29.6
	Dalian	73	31.6	26.3	43.4	42.6

**Table 9:** Cube compressive strength loss rate.

**Specimen Number**	**NC**	**DSC-20**	**DSC-40**	**DSC-60**
strength loss rate/%	19.41	36.13	17.48	16.21

## Data Availability

The original contributions presented in this study are included in the article. Further inquiries can be directed to the corresponding authors.

## References

[1] Wang R.J., Hu Z.Y., Li Y., Wang K., Zhang H. (2022). Review on the deterioration and approaches to enhance the durability of concrete in the freeze-thaw environment. Constr. Build. Mater..

[2] Padmakumar G.P., Srinivas K., Uday K.V., Iyer K.R., Pathak P., Keshava S.M., Singh D.N. (2012). Characterization of aeolian sands from Indian desert. Eng. Geol..

[3] Bendixen M., Best J., Hackney C., Iversen L.L. (2019). Time is running out for sand. Nature.

[4] Elipe M.G.M., López-Querol S. (2014). Aeolian sands: Characterization, options of improvement and possible employment in construction—The State-of-the-art. Constr. Build. Mater..

[5] Yan W., Wu G., Dong Z. (2019). Optimization of the mix proportion for desert sand concrete based on a statistical model. Constr. Build. Mater..

[6] Li Z., Gan D. (2022). Cyclic behavior and strength evaluation of RC columns with dune sand. J. Build. Eng..

[7] Li Z., Ma R., Li G. (2020). Experimental Study on the Shear Strength of Dune Sand Concrete Beams. Adv. Civ. Eng..

[8] Li Z., Zhai D., Li J. (2022). Seismic behavior of the dune sand concrete beam-column joints under cyclic loading. Structures.

[9] Zhang S.L., Yuan K., Zhang J.M., Guo J.L. (2020). Experimental Study on Performance Influencing Factors and Reasonable Mixture Ratio of Desert Sand Ceramsite Lightweight Aggregate Concrete. Adv. Civ. Eng..

[10] Li Y.G., Zhang H.M., Liu G.X., Hu D.W., Ma X.R. (2020). Multi-scale study on mechanical property and strength prediction of aeolian sand concrete. Constr. Build. Mater..

[11] Li Y., Zhang H., Liu X., Liu G., Hu D., Meng X. (2019). Time-Varying Compressive Strength Model of Aeolian Sand Concrete considering the Harmful Pore Ratio Variation and Heterogeneous Nucleation Effect. Adv. Civ. Eng..

[12] Zhang W., Zheng M.L., Zhu L.L., Lv Y.Z. (2022). Mix design and characteristics evaluation of high-performance concrete with full aeolian sand based on the packing density theory. Constr. Build. Mater..

[13] Zhou Y., Li H., Yu S.Y., Guo H.L. (2024). Experimental Investigation of the Impact of Blended Fibers on the Mechanical Properties and Microstructure of Aeolian Sand Concrete. Materials.

[14] Jiang L., Niu D., Yuan L., Fei Q. (2015). Durability of concrete under sulfate attack exposed to freeze-thaw cycles. Cold Reg. Sci. Technol..

[15] Tian J., Wang W., Du Y. (2016). Damage behaviors of self-compacting concrete and prediction model under coupling effect of salt freeze-thaw and flexural load. Constr. Build. Mater..

[16] Wang X., Shen X., Wang H., Gao C. (2015). Nuclear magnetic resonance analysis of concrete-lined channel freeze-thaw damage. J. Ceram. Soc. Jpn..

[17] Wang L., Xiao W., Wang Q., Jiang H.L., Ma G.W. (2022). Freeze-thaw resistance of 3D-printed composites with desert sand. Cem. Concr. Compos..

[18] Zhang H., Wang D., Jing P. (2023). Study on freeze-thaw damage characteristics of aeolian sand concrete with different replacement ratios. J. Henan Univ. Sci. Technol. (Nat. Sci.).

[19] Dong W., Shen X.-D., Xue H.-J., He J., Liu Y. (2016). Research on the freeze-thaw cyclic test and damage model of Aeolian sand lightweight aggregate concrete. Constr. Build. Mater..

[20] Bai J., Zhao Y., Shi J., He X. (2022). Damage degradation model of aeolian sand concrete under freeze-thaw cycles based on macro-microscopic perspective. Constr. Build. Mater..

[21] (2011). Specification for Mix Proportion Design of Ordinary Concrete.

[22] (2013). Standard Specification for Concrete Aggregates.

[23] (2010). Standard for Test Method of Long-Term Performance and Durability of Ordinary Concrete.

[24] Powers T.C. (1945). A working hypothesis for further studies of frost resistance of concrete. J. Am. Concr. Inst..

[25] Powers T.C., Helmuth R.A. (1953). Theory of Volume Changes in Hardened Portland Cement Paste During Freezing. Highw. Res. Board Proc..

[26] Liu H., Liu Y., Jiang Y., Che J., Yang W. (2023). Study on frost resistance of desert sand concrete under freeze-thaw cycle. Ind. Constr..

[27] Li Y., Zhang H., Chen S., Wang H., Liu G. (2022). Multi-scale study on the durability degradation mechanism of aeolian sand concrete under freeze-thaw conditions. Constr. Build. Mater..

[28] Hamada H.M., Abed F., Al-Sadoon Z.A., Elnassar Z., Hassan A. (2023). The use of treated desert sand in sustainable concrete: A mechanical and microstructure study. J. Build. Eng..

[29] Li G.F., Shen X.D. (2019). A Study of the deterioration law and mechanism of aeolian-sand powder concrete in the coupling environments of freeze-thaw and carbonization. J. Ceram. Soc. Jpn..

[30] Zhang G., Geng T., Lu H., Wang M., Li X. (2021). Damage Model of Desert Sand Fiber Reinforced Concrete under Freeze-Thaw Cycles. Bull. Chin. Ceram. Soc..

[31] Wang C., Liu L., Cao F., Chen X., Ni L., Zhang Z. (2020). Experimental study on mechanical properties of recycled concrete after freeze-thaw cycles. J. Build. Struct..

[32] Gan L., Liu Y., Shen Z., Chen G. (2023). Damage evolution law of concrete under sulfate attack and freeze-thaw cycle. J. Huazhong Univ. Sci. Technol. (Nat. Sci. Ed.).

[33] Liu Y. (2020). Experimental Study on Mechanical Properties and Durability of Aeolian sand Concrete.

[34] (2019). Standard for Design of Concrete Structure Durability.

[35] Li J., Peng X., Deng Z., Cao J., Guan Y., Lin L., Tian J., Li F., Wang A., Wang Z. (2000). Quantitative Design on the Frost-resistance of Concrete. Concrete.

[36] Wu H., Jin W., Yan Y., Xia J. (2012). Environmental zonation and life prediction of concrete in frost environments. J. Zhejiang Univ. (Eng. Sci.).

